# Differential effect of acute *versus* persistent insect-specific flavivirus infection on superinfection exclusion of West Nile, Zika and chikungunya viruses in RNAi-competent and -deficient mosquito cells

**DOI:** 10.1016/j.onehlt.2024.100960

**Published:** 2024-12-24

**Authors:** Wessel Willemsen, Nick Helmes, Gijs J. Overheul, Marleen Henkens, Ruben Spruijt, Ronald P. van Rij, Monique M. van Oers, Gorben P. Pijlman, Jelke J. Fros

**Affiliations:** a Laboratory of Virology, Wageningen University and Research, Wageningen, the Netherlands; b Department of Medical Microbiology, Radboud University Medical Center, Nijmegen, the Netherlands

**Keywords:** Insect-specific flavivirus, Superinfection exclusion, Flavivirus, Binjari virus, Zika virus, West Nile virus, Chikungunya virus, C6/36, Aag2, Mosquito cells, Cytopathic effects, Acute infections, Persistent infections, RNAi

## Abstract

Millions of people are annually infected by mosquito-transmitted arboviruses including dengue virus (DENV), West Nile virus (WNV), Zika virus (ZIKV) and chikungunya virus (CHIKV). Insect-specific flaviviruses (ISFs), which only infect mosquitoes and cannot replicate in vertebrates, can offers a potential one health strategy to block the transmission of arboviruses by reducing the mosquito's susceptibility for subsequent arbovirus infections through superinfection exclusion (SIE),. Most SIE studies focus on acute ISF infections in RNAi-deficient *Aedes albopictus* C6/36 cells. Because ISFs are known to persistently infect mosquitoes, acute infections in C6/36 cells may not accurately reflect natural interactions between ISFs and arboviruses. To study the underlying mechanisms for SIE, we persistently infected C6/36 and RNAi-competent *Aedes aegypti* Aag2 cells with the ISF Binjari virus (BinJV) and a BinJ-ZIKV chimera that contains the ZIKV prME structural genes. SIE of WNV, ZIKV and CHIKV by BinJV was more pronounced in acute than in persistently infected cells and much stronger in acutely infected C6/36 cells compared to Aag2 cells. The viability of RNAi-deficient mosquito cells was severely reduced upon acute ISF infection, which correlated to the observed SIE. However, persistently infected mosquito cells still inhibited subsequent arbovirus replication. Moreover, RNAi-competent Aag2 cells were better protected against ZIKV superinfection when they were pre-infected with BinJ-ZIKV as compared to BinJV. Therefore, acute ISF infections and strong cytopathic effects in RNAi-deficient cells augment SIE, while in persistently infected cells SIE is established through RNAi-dependent and independent mechanisms. This highlight the importance of using more representative *in vitro* models.

## 1 Introduction

Arthropod-borne viruses (arboviruses) are transmitted between humans and vertebrate animals by hematophagous arthropod vectors such as mosquitoes, ticks and biting midges. After the arthropod vector has taken an infectious blood meal, the arbovirus infects the vector, replicates and finally localizes in the salivary glands before the vector is able to transmit the virus to the next vertebrate host. Arboviruses are a global health burden and collectively responsible for infecting more than 400 million people annually, with potential fatal outcomes [1]. Many clinically significant arboviruses belong to the genus *Orthoflavivirus* (Family *Flaviviridae),* which includes dengue virus (DENV), Zika virus (ZIKV) and West Nile virus (WNV).

Besides the many dual-host arboviruses, the genus *Orthoflavivirus* also contains vertebrate viruses with no known vectors as well as insect-specific flaviviruses (ISFs) [2]. ISFs are incapable of infecting vertebrate hosts and their host range is therefore limited to invertebrate species. The ISFs comprise two phylogenetically distinct groups, the classical insect-specific flaviviruses (cISFs) also named lineage I ISFs and the dual-host affiliated insect-specific flaviviruses (dISFs) or lineage II ISFs. The latter group is phylogenetically closely related to dual-host mosquito-borne flaviviruses (MBFVs) (reviewed in [3]). Because of their inability to infect vertebrate cells, these ISFs have garnered only limited interest in the past. However, in recent years the perspective has changed with the discovery that some ISFs can suppress the replication of medically important flaviviruses in co-infected mosquito cells *via* a process called superinfection exclusion (SIE) [[4], [5], [6], [7], [8]].

Traditionally, investigations into the *in vitro* dynamics between ISFs and MBFVs have predominantly been studied in the *Aedes albopictus* mosquito cell line C6/36 during acute ISF infection [5,7,8]. In mosquitoes, the primary antiviral responses rely on small non-coding RNAs that can silence complementary viral RNAs (reviewed in [9]) which consist out of three classes of RNA, including microRNAs (miRNAs) (∼22–23 nts), small-interfering RNAs (siRNAs)(21 nts), and PIWI-interacting RNAs (piRNAs) (25–30 nts) (reviewed in [10]). C6/36 cells have dysfunctional antiviral RNA interference (RNAi) response as these cells are incapable of generating short-interfering RNAs (siRNAs) due to a defective Dicer-2 (Dcr-2) gene [11,12]. This has made C6/36 cells a valuable tool for studying viral replication dynamics because of their high susceptibility to virus infection. Furthermore, C6/36 cells have been instrumental in the discovery of ISFs due to the cytopathic effects (CPE) generally observed upon their infection. The manifestation of CPE in C6/36 upon infection differs between ISFs from mild to more severe CPE and can be recognised by one or more of the following: growth inhibition, cell rounding, syncytia development, detachment of the monolayer, cell aggregation, cell lysis and death [8,[13], [14], [15], [16], [17]]. In sharp contrast, under natural conditions, ISFs persistently infect vectors and RNAi-competent mosquito cells without notable fitness loss or effects on cell viability [[17], [18], [19]]. When ISF-induced CPE in C6/36 cells affects cellular homeostasis and viability, this will likely impact viral replication dynamics of a superinfecting arbovirus. Therefore, the development of CPE in C6/36 cells during acute ISF infections can complicate the interpretation of SIE experiments. Arguably, the outcomes of such studies may not accurately reflect how natural, persistent ISF infections in Dcr-2 competent mosquito cells would affect subsequent superinfection with clinically significant arboviruses.

To address these complexities, this study assesses how acute and persistent ISF infections in mosquito cells differentially affect viral replication and SIE of dual-host flaviviruses ZIKV and WNV and the unrelated alphavirus chikungunya virus (CHIKV) (Family *Togaviridae*). Furthermore, the contribution of a functional RNAi response in SIE is investigated. To this end, we generated C6/36 and RNAi-competent *Aedes aegypti* Aag2 cells that are persistently infected with the dISF Binjari virus (BinJV). BinJV was originally isolated from *Aedes normanensis* in northern Australia and is exceptionally tolerant to the exchange of its structural proteins with those of MBFVs such as ZIKV [[20], [21], [22]]. A BinJV/ZIKV-prME chimera (BinJ-ZIKV) was also included in this study to test the importance of sequence homology between the ISF and the superinfecting arbovirus for induction of SIE. This study provides novel information on distinct RNAi-dependent and independent factors that contribute to ISF-induced SIE and proposes improved *in vitro* models to study SIE under more representative conditions.

## 2 Materials and methods

### 2.1 Viruses and cells

*Aedes albopictus* C6/36, *Aedes aegypti* Aag2 [23] and Aag2 Ago2-deficient cells (g4#1, described below) were all cultured as a monolayer in Leibovitz L-15 medium (Gibco, Carlsbad, CA, USA) supplemented with 10 % heat inactivated fetal bovine serum (FBS; Gibco), 2 % tryptose phosphate broth (Gibco) and 1 % nonessential amino acids (Gibco) at 27 °C. The Aag2 and Aag2 Ago2 deficient cells were previously cleared from insect-specific viruses Phasi-charoen-like virus (PCLV) and cell-fusing agent virus (CFAV) [23].

African green monkey kidney Vero E6 cells were cultured as a monolayer in Dulbecco's Modified Eagle Medium (DMEM; Gibco) supplemented with heat inactivated 10 % FBS, and Penicillin streptomycin (100 μg/ml; Sigma-Aldrich) (P/S). Vero cells were cultured at 37 °C and 5 % CO_2_. Prior to virus infections, Vero cells were seeded in HEPES-buffered DMEM medium (Gibco) supplemented with 10 % FBS and P/S.

A passage 3 stock of BinJV (Genebank ID: MG587038) and a passage 1 stock of BinJ-ZIKV (chimeric BinJV encoding prME genes of ZIKV_Natal_, amino acid Ala123 to Ala794; Genebank ID: KU527068 with the rest of the genome derived from BinJV) [21] were grown on either C6/36 or Aag2 cells and harvested at 4 dpi. A passage 3 stock of WNV Greece 2010 (GenBank KC496015.1) and a passage 6 stock of ZIKV, Suriname 2016 (GenBank accession no. KU937936.1; EVAg Ref-SKU 011 V-01621; obtained from the Erasmus Medical Center, Rotterdam, the Netherlands) were grown on African green monkey kidney (Vero) cells and harvested 3 dpi. Finally, a passage 3 stock of CHIKV 37997 (Genbank ID: AY726732) was grown on C6/36 cells and harvested 3 days post infection. All handling of infectious ZIKV, WNV and CHIKV was performed in the biosafety level 3 laboratory at Wageningen University and Research.

### 2.2 CRISPR/Cas9

The pPUb-Cas9-AaegU6.2 plasmid was generated by replacing the *D. melanogaster* actin 5C and U6 promoters in pAc-sgRNA-Cas9 (provided by Ji-Long Liu; Addgene #49330) with the *A. aegypti* polyubiquitin and U6.2 (AAEL028848) promoters, respectively using In-Fusion cloning (Takara). Four single guide sequences targeting Ago2 (AAEL017251) were cloned directly downstream of the U6.2 promoter into the *Sap*I restriction site. Oligonucleotide sequences are listed in table S1.

Aag2 C3PC12 cells [23] were transfected with pPUb-Cas9-AaegU6.2 containing one of the Ago2 sgRNAs using X-tremeGENE HP transfection reagent (Roche). The next day, the cells were treated with puromycin (InvivoGen) at a concentration of 4 μg/ml and split at a 1:2 ratio in the presence of puromycin after a 24-h incubation. The rest of the cells were used for genomic DNA isolation using the Quick-DNA miniprep kit (Zymo Research) and PCR (Promega) to analyze the cleaving efficiency of the different guides on agarose gel. At 5 days after transfection, cells that were transfected with the most effective guides were seeded as single cells in 96-well plates without puromycin. After 6 weeks, the clones that grew out were further expanded and the Ago2 sequence was analyzed by genomic DNA isolation, PCR using primers flanking the targeted region, and Sanger sequencing. Cells treated with guide 1 (clone #4) and guide 4 (clone #1) contained out-of-frame deletions in the first exon of Ago2 leading to premature stop codons. Cell clone g4#1 was used for virus infection experiments.

RNAi efficiency was analyzed in Ago2 knockout cells and parental wildtype Aag2 C3PC12 cells using a luciferase based RNAi reporter assay, akin to the one described in a study from Cleef et al., 2014 [24]. Double-stranded RNA (dsRNA) was produced by *in vitro* transcription of T7 promoter-flanked PCR products generated by PCR using the primers listed in Table S1. Cells were transfected using X-tremeGENE HP with plasmids pPUb-Fluc (encoding firefly luciferase), pPUb-Rluc (encoding Renilla luciferase) and dsRNA targeting GFP as control or firefly luciferase. The luciferase plasmids were generated by removing the Gateway cassette in pUGW and replacing EGFP with either Fluc or Rluc using restriction cloning and the primers in Table S1. At 2 days after transfection, the cells were harvested to measure the luminescence using the Dual-Luciferase Reporter Assay System (Promega). The Fluc counts were normalized to Rluc counts and the fold silencing was calculated relative to the dsGFP control.

### 2.3 Generation of BinJV/BinJ-ZIKV persistently infected cell-lines

C6/36 and Aag2 cells were seeded at 60 % confluency in T25 flasks and infected with either BinJV or BinJ-ZIKV at a MOI of 0.1 in 2 ml of Leibovitz L-15 medium. After two hours, the inoculum was removed and 4 ml of fresh medium was added. Cells were passaged 2 times a week by mechanical detachment when they were fully confluent after 3 or 4 days. Periodically 50 μl of culture was harvested and stored at −80 °C to be able to determine the virus titer by end point dilution assay (EPDA) on C6/36 cells at a later time.

### 2.4 Immunofluorescence assay

Aag2 and C6/36 cells were seeded in 24-well plates at 150,000 and 600,000 cells per well, respectively. Cells were fixed with 4 % formaldehyde in PBS for 15 min at room temperature. Monolayers were washed three times with PBS and subsequently permeabilized with 0.1 % sodium dodecyl sulphate (SDS) for 10 min. Cells were blocked using 1 % skimmed milk powder (ELK, Campina, Eindhoven, the Netherlands) in PBS. Primary antibodies (BJ-6E6, hybridoma supernatant diluted 1:3 [22] and 4G2, mouse monoclonal; 1:100 dilution [25]) in 1 % ELK in PBS were used for immune-labelling of BinJV and ZIKV viral E antigen, respectively, for 1 h. After three PBS washes the second antibody (goat anti-mouse Alexa-Fluor™ 488 1:2000 diluted in 1 % ELK in PBS) was added and cells were further incubated for 45 min. Cells were washed again three times with PBS and the nucleus was stained using Hoechst (1:100 in PBS) for 2 min. Lastly cells were washed three times with PBS and visualized with a Zeiss Observer Z.1 fluorescence microscope.

### 2.5 Viral growth curves

Triplicate cultures of Aag2 and C6/36 cells were simultaneously seeded in 24 wells-plates at 150,000 and 600,000 cells per well, respectively. Cells were infected with BinJV or BinJ-ZIKV at an MOI of 1. After three days, three extra wells for each cell line were detached and counted to determine the number of cells per well after the 3-day incubation period. Triplicate cultures of uninfected or persistently infected Aag2 and C6/36 were seeded according to the determined cell number after which they and the BinJV or BinJ-ZIKV infected cells were infected with ZIKV, WNV or CHIKV at an MOI of 1. After 1 h, cells were washed twice with PBS and fresh medium was added. Supernatant samples were collected each day from day 0 to day 4 and stored at −80 °C, and later titrated by EPDA on Vero cells.

### 2.6 Virus titrations

WNV, ZIKV and CHIKV were titrated on Vero cells while BinJV and BinJ-ZIKV were titrated on C6/36 cells. Vero cell suspensions were prepared by detaching Vero cells from a T25 flask with 3 ml of Trypsin-EDTA (Gibco), after which 9 ml of culture medium was added. C6/36 cell suspensions were prepared by mechanically detaching the monolayer of a T25 with a glass pipette in 4 ml of Leibovitz cell culture medium. Supernatant samples were thawed, vortexed and serial dilutions were made in the respective cell culture media for titrations on Vero or C6/36 cells. Next, vero and C6/36 cell suspensions were added to the virus dilutions in a 1:1 ratio. Ten microliters from these mixtures were plated in 6-fold in micro-titer plates (Nunc, Roskilde, Denmark). EPDAs of samples infected with WNV, ZIKV or CHIKV were scored at 4 dpi based on virus induced CPE. EPDAs of samples infected with BinJV or BinJ-ZIKV were scored at 7 dpi based on virus induced CPE.

### 2.7 Small RNA analysis

Monolayers of Aag2 and C6/36 cells were plated in 24-well plates at 150,000 and 600,000 cells per well, respectively, and infected with BinJ-ZIKV at an MOI of 1. Three extra wells for each cell line were detached and counted to determine the number of cells per well after 2-day incubation so BinJ-ZIKV persistently infected Aag2 and C6/36 could be density matched. At three days post BinJ-ZIKV infection, RNA from acutely and persistently infected Aag2 and C6/36 cells was isolated using TRIzol reagent (Invitrogen) following the manufacturer's protocol. Small RNA libraries were generated from ∼1 μg total RNA on a DNBSEQ sequencing platform (BGI Group, Shenzhen, Guangdong, China). Trimmed single-end FASTQ reads were generated with an in-house filtering protocol of BGI. Small RNA sequencing libraries were analyzed on the Galaxy webserver as described previously [26]. Reads were mapped to the viral genome of BinJ-ZIKV with Bowtie2 version 2.5.3 + galaxy0 allowing 1 mismatch with a seed length of 28. Size distributions of small RNAs and genome distributions of siRNAs and piRNAs were produced with in house R scripts (Supplementary Code S1). Plots were generated in Graphpad Prism 10.2.2. Sequence signatures were generated from mapped sense and reverse-complemented antisense reads of length 25–30 nt that were 3’trimmed to 20 nt using Weblogo3.

### 2.8 MTT assay

Cell viability was assessed using the MTT reduction assay [27]. Briefly, monolayers of Aag2 and Aag2 Ago2 KO and C6/36 were plated in 24-well plates at 150,000, 150,000 and 600,000 cells per well respectively after which they were infected with BinJV and BinJ-ZIKV at an MOI of 1 or mock infected. At 4 dpi, 50 ul of MTT was added to each well resulting in a final concentration of 0.5 mg/ml. The cells were incubated for 3 h at 28 °C, protected from light. Medium was aspirated and the formed formazan salts were dissolved in 1 ml of dimethyl sulfoxide for 15 min at 37 °C. The OD_490_ was obtained from each well using a plate reader (TECAN, Switzerland). Uninfected wells were used as negative control with their OD values set at 100 % relative viability.

### 2.9 Statistics

To determine differences in viral titers between control virus groups and dual superinfected groups, a two-way ANOVA followed by a Tukey test for correction of multiple comparisons was used on all SIE experiments. A one-way ANOVA was used for determining the differences in viability between mock infected cells and infected cells for the MTT assay. All statistics were performed in GraphPad Prism version 10.2.2.

## 3 Results

### 3.1 The insect-specific flavivirus Binjari virus persistently infects *Aedes* mosquito cell lines

**Fig. 1:**
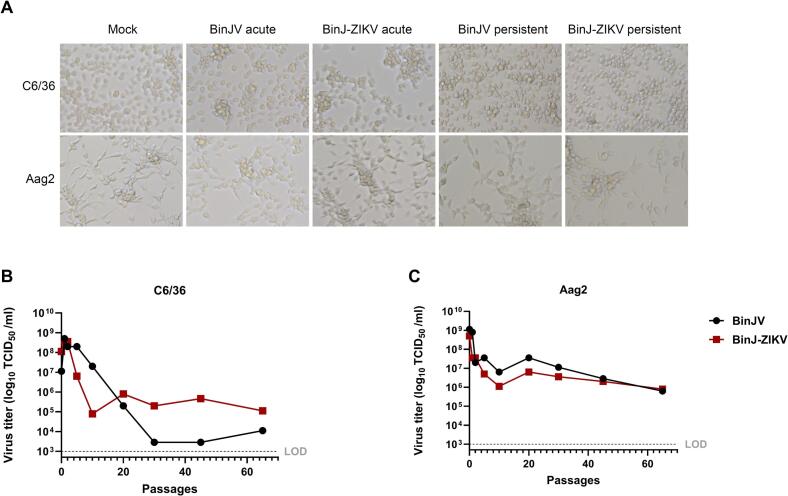
Establishment and characterisation of C6/36 and Aag2 mosquito cell lines persistently infected with BinJV and BinJ-ZIKV. **(A)** Brightfield pictures of *Aedes albopictus* C6/36 and *Aedes aegypti* Aag2 cells mock-infected and acutely (MOI 1, 3 dpi) or persistently infected with BinJV or BinJ-ZIKV (Images were taken at passage 67, except for C6/36 cells persistently infected with BinJV, which were taken at passage 55). Virus titers of the supernatant of (persistently) infected **(B)** C6/36 cells and **(C)** Aag2 cells with BinJV or BinJ-ZIKV determined by EPDA. The limit of detection (LOD) for BinJV and BinJ-ZIKV was 1 × 10^3^ TCID_50_/ml.

**Supplementary Fig. 1:**
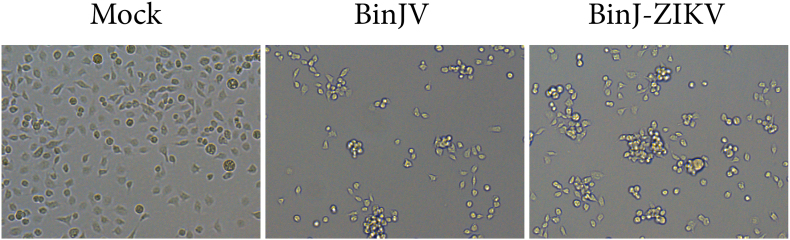
**Clustered cell growth of C6/36 cells after BinJV or Binj-ZIKV infection**. Brightfield pictures of C6/36 cells showing normal growth of mock infected C6/36 cells compared to growth from clusters of cells after BinJV or BInj-ZIKV infection (MOI 0.1; 7 dpi)

To generate persistently infected mosquito cells, C6/36 and Aag2 cells were infected with BinJV or BinJ-ZIKV at an MOI of 0.1. During acute infections, as early as 3 days post infection (dpi), the C6/36 cell cultures infected with either virus showed growth inhibition and CPE-related changes in cell morphology with cell clumping and some detachment, leading to reduced confluency of the monolayer (Fig. 1A, top). There was no cell growth until 7 dpi, after which small clusters of cells were observed to grow (Fig. S1). Four days later (11 dpi) both BinJV and BinJ-ZIKV infected C6/36 cultures reached confluency and were passaged. After 5 passages no differences were seen in growth rates and morphology compared to non-infected cells, and CPE was no longer observed. In contrast, Aag2 cells displayed no morphological changes or distinguishable CPE during the acute phase of BinJV or BinJ-ZIKV infection until the first cell passage. Moreover, the growth rate of infected Aag2 cell lines showed no differences compared to the non-infected cells, and the infected cells were passaged at 4 dpi (Fig. 1A, bottom).

All four infected cell lines were passaged twice a week for a minimum of 65 passages. Periodically, cell culture medium from the infected cell lines was used to perform a titration on uninfected C6/36 cells using an EPDA. During the acute phase of infection, the cell culture media of C6/36 cells infected with BinJV and BinJ-ZIKV reached peak viral titers of 5.0 × 10^8^ and 3.6 × 10^8^ TCID_50_/ml, respectively. Shortly thereafter, the infectious viral titers of both viruses decreased with each passage of the infected C6/36 cells. The viral titer of the BinJV-infected C6/36 cell line stabilized after 30 passages ranging from 2.9 × 10^3^ to 1.1 × 10^4^ TCID_50_/ml (Fig. 1B). In comparison, the infectious viral titers produced by the BinJ-ZIKV-infected C6/36 cells stabilized after 10 passages with titers ranging from 8.0 × 10^4^ to 8.0 × 10^5^ TCID50/ml (Fig. 1B). Therefore, the amount of infectious virus produced by C6/36 cells persistently infected with BinJV and the BinJ-ZIKV chimera was decreased by 1.8 × 10^5^-fold and 4.5 × 10^3^-fold, respectively, compared to the initial acute infection.

In Aag2 cells, BinJV and BinJ-ZIKV infections initially produced peak viral titers of 1.1 × 10^9^ and 5.0 × 10^8^, respectively. The infectious viral titers for both viruses then decreased rapidly until stabilizing after 10 passages, with average titers fluctuating between 6.3 × 10^5^ and 3.6 × 10^7^ TCID_50_/ml (Fig. 1C). Compared to the initial acute infection, this represents a drop in viral titers of up to 1.8 × 10^3^-fold and 6.3 × 10^2^-fold for BinJV and BinJ-ZIKV, respectively.

This indicates that both Aag2 and C6/36 cell lines can support persistent replication of BinJV and the BinJ-ZIKV chimera with strongly reduced viral loads and the absence of visible virus-induced CPE, with the latter a stark contrast to acute infections in C6/36.

**Fig. 2:**
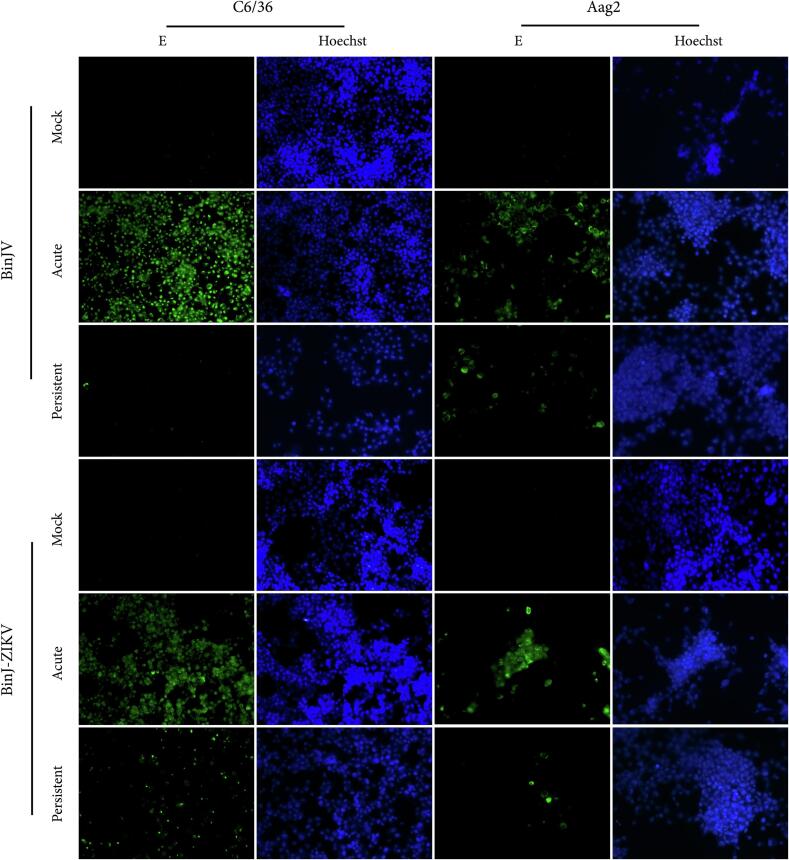
BinJV & BinJ-ZIKV viral E protein expression in persistently infected C6/36 and Aag2 cells. Fluorescent microscope pictures of C6/36 and Aag2 cells mock-infected and acutely (MOI 1, 3 dpi) or persistently infected with BinJV or BinJ-ZIKV. Viral E protein was visualized with 6E6 and 4G2 antibodies for BinJV and BinJ-ZIKV, respectively (green). Nuclei were counterstained using Hoechst (blue). (For interpretation of the references to colour in this figure legend, the reader is referred to the web version of this article.)

To investigate whether the observed reduction in infectious virus titres in persistently infected cell lines corresponds to reduced viral protein expression, an immunofluorescence assay (IFA) was performed to visualise the BinJV and ZIKV envelope proteins (E) (Fig. 2). Primary antibody BJ-6E6 [22] and 4G2 (pan-flavivirus α-E) [25] were used for the visualisation of the BinJV E protein and ZIKV E protein, respectively. As a positive control, naïve Aag2 and C6/36 cells were acutely infected with BinJV and BinJ-ZIKV and immuno-labelled at 3 dpi. In these acute infections almost every cell displayed fluorescence, indicating the presence of viral E protein. In the persistently infected cell lines, viral E proteins were only observed in a small percentage of cells. In particular, BinJV exhibited a near absence of positive cells in the C6/36 cells compared to BinJ-ZIKV Notably, more cells were expressing viral E protein in the persistently infected Aag2 cells compared to the C6/36 cells. Overall, these results indicate reduced expression of viral E protein in persistently infected cells, which in most cells likely remains below the detection limit of the IFA.

### 3.2 *In vitro* superinfection exclusion of arboviruses is dependent on ISF infection state and cell line

Previous studies have repeatedly shown that acute infections in C6/36 cells with ISFs including BinJV can limit arbovirus replication [4,5,8,28]. To investigate whether SIE can also occur in Aag2 cells and whether persistent BinJV replication also interferes with the replication of medically important arboviruses, the replication of ZIKV, WNV, and CHIKV was assessed in uninfected, acutely infected and persistently infected Aag2 or C6/36 cells. First, naïve Aag2 and C6/36 cells were infected with BinJV at an MOI of 1 to generate acutely infected cells. Three days post infection, cells persistently infected with BinJV (described above) and uninfected cells were seeded in a density that matched the acutely infected samples. Next, these uninfected, BinJV acutely and persistently infected C6/36 and Aag2 cells were (super)infected with ZIKV, WNV or CHIKV at an MOI of 1. Cell culture medium samples were taken daily and titrated on vertebrate Vero cells that only support the replication of the dual-host arboviruses, and not of the ISF.

**Fig. 3:**
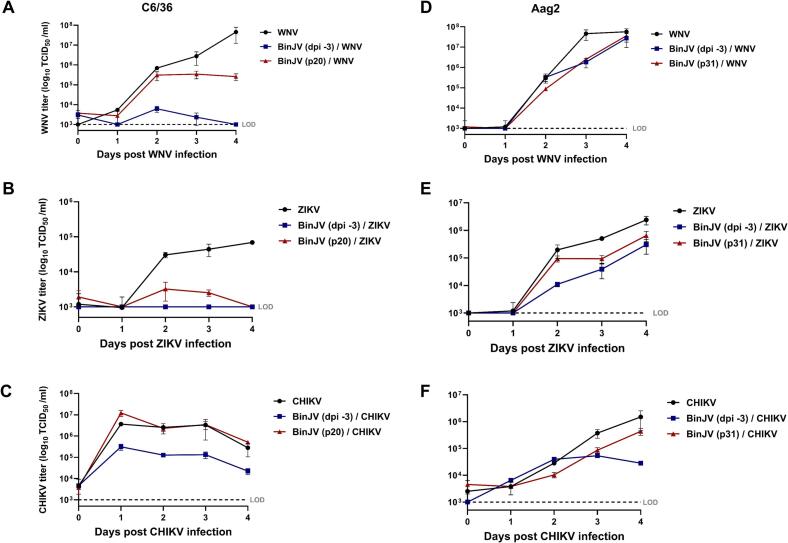
SIE of acute or persistent BinJV infection on ZIKV, WNV & CHIKV in Aag2 and C6/36 cells. C6/36 cells **(A-C)** and Aag2 cells **(D—F)** were either not infected, acutely infected (MOI 1) or persistently infected with BinJV prior to superinfection with WNV **(A,D)**, ZIKV **(B,E)** or CHIKV **(C,F)** at an MOI of 1**.** Supernatant was collected on the indicated days and viral titers were determined using EPDA. Shown are the mean virus titers and SEM from 3 biological replicates. Dotted line represents the limit of detection (LOD) for ZIKV, WNV and CHIKV at 1 × 10^3^ TCID_50_/ml.

In C6/36 cells that were acutely infected with BinJV, ZIKV, WNV, and CHIKV replicated to significantly lower titers compared to infections in cells that were not previously infected with BinJV (Fig. 3A,B,C). Notably, the replication of ZIKV during superinfection was significantly impacted as the ZIKV titers never surpassed the limit of detection (LOD) of 1 × 10^3^ TCID_50_ /ml compared to 7 × 10^4^ TCID50/ml in cells that were not previously infected with BinJV (*p* < 0.0001). The WNV titers in the acutely infected C6/36 cells were only above the LOD at 2 and 3 dpi with a significant reduction in virus titer of at least 50,000-fold on day 4 (*p* < 0.01). CHIKV titers during superinfection also displayed a reduction in titer compared to a CHIKV infection on uninfected C6/36 cells albeit much lower with a 12-fold reduction 1 dpi (*p* < 0.0001). Interestingly, the persistently infected C6/36 cells showed higher titers for ZIKV, WNV and CHIKV compared to the superinfected cells acutely infected with BinJV. CHIKV titers from C6/36 cells persistently infected with BinJV showed similar titers to the control that was not infected with BinJV.

Importantly, SIE of ZIKV and WNV was less pronounced or even absent in Aag2 cells, which have a functional RNAi response (Fig. 3D,E,F). Superinfection of WNV on Aag2 cells that were either acutely or persistently infected with BinJV resulted in similar peak WNV titres at 4 dpi compared to WNV infection in the absence of BinJV. ZIKV titers in Aag2 cells that were acutely or persistently infected with BinJV were significantly reduced 4-fold (*p* < 0.0001) and 8-fold (*p* < 0.0001), respectively, at 4 dpi. Surprisingly, BinJV inhibited the replication of CHIKV to a greater extent in Aag2 cells compared to the C6/36 cells. Overall, we observed stronger inhibition of arbovirus replication in cells that were acutely infected with BinJV compared to persistently infected cells. This phenotype was exacerbated in C6/36 cells, which also showed strong CPE during acute BinJV infections (see Fig. 1A).

### 3.3 Sequence homology between BinJ-ZIKV and ZIKV promotes superinfection exclusion in Aag2 cells

To investigate to what extent RNAi can play a role in SIE, the *in vitro* replication of ZIKV and WNV was assessed when superinfected on Aag2 cells that were initially infected with BinJV or the BinJ-ZIKV chimera. ZIKV and the BinJ-ZIKV share a 2 kb homologous region encoding the ZIKV prME structural proteins. To further investigate sequence similarity between BinJV and either virus (WNV and ZIKV), discontingous megablast was used. Alignment of both viruses resulted in a 25 % query cover for WNV with 65.54 % identity and a 23 % query cover for ZIKV with 66.13 % identity. These results indicate that the overall sequence similarity between BinJV and these two viruses is equally low. To assess this interaction in more detail, Aag2 cells were seeded and infected with BinJ-ZIKV. After 3 days, BinJV and BinJ-ZIKV persistently infected cells and uninfected cells were density matched with the acutely infected samples and superinfected with ZIKV or WNV.

**Fig. 4:**
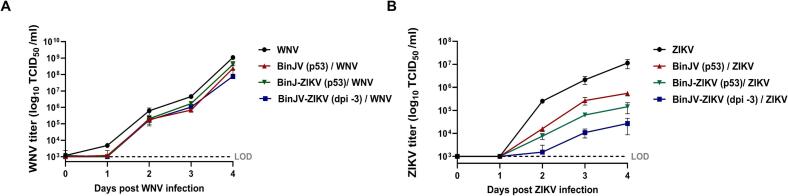
SIE of ZIKV and WNV by BinJ-ZIKV in Aag2 cells. **(A)** WNV and **(B)** ZIKV titers after infections in Aag2 cells acutely (MOI 1) infected with BinJV or persistently infected with BinJV or BinJ-ZIKV. Supernatant was collected on the indicated days and viral titers were determined using EPDA. Shown are the mean virus titers and SEM from 3 biological replicates. Dotted line represents the limit of detection (LOD) for ZIKV, WNV and CHIKV at 1 × 10^3^ TCID_50_/ml.

Superinfection of WNV on cells infected with BinJV or the BinJ-ZIKV chimera resulted in reduced WNV replication compared to WNV replication on cells uninfected by BinJV. Compared to Aag2 cells that were not infected with BinJV, the BinJ-ZIKV persistently infected cells displayed the smallest reduction in WNV titers, followed by BinJV persistently infected cells and BinJ-ZIKV acutely infected cells (Fig. 4A). However, these differences were not statistically significant. These effects were also much smaller than the effects that BinJV and BinJ-ZIKV replication had on superinfecting ZIKV replication, similar to what was observed in Fig. 3.

ZIKV replication was significantly decreased in all virus infected groups compared to the mock that was not infected by BinJV or BinJ-ZIKV (*p* < 0.0001). Similar to the previous experiment with BinJV (Fig. 3) cells acutely infected with BinJ-ZIKV inhibited the replication of superinfecting ZIKV more than cells that were persistently infected with the BinJ-ZIKV chimera. Interestingly, a notable difference in the reduction of ZIKV titers was observed between the two persistently infected cell lines. ZIKV superinfection on the BinJ-ZIKV persistently infected cell line resulted in a further 4-fold reduction in ZIKV titers compared to a ZIKV superinfection on the BinJV persistently infected cell line (Fig. 4B). This result suggests that the sequence homology between the BinJ-ZIKV chimera and ZIKV further reduces ZIKV replication during superinfection compared to superinfections on cells that are infected with BinJV without sequence homology.

### 3.4 Differential small RNA responses to BinJ-ZIKV across different infection states and mosquito cells

To investigate whether and to what extent the acutely and persistently BinJ-ZIKV infected cells harboured an effective siRNA response to both BinJV and ZIKV sequences, RNA was isolated from BinJ-ZIKV acutely and persistently infected Aag2 and C6/36 cells and subjected to small RNA deep sequencing. The acutely infected Aag2 and C6/36 cells contained high BinJ-ZIKV titers (6.3 × 10^7^ and 4.6 × 10^7^ TCID_50_/mL, respectively) 3 dpi, compared to persistently infected cells (8.0 × 10^5^ and 4.6 × 10^5^ TCID_50_/mL, respectively). Small RNA reads were then mapped to the BinJ-ZIKV genome. Mapped reads were normalized to the total number of reads and size-distributions were generated.

**Fig. 5:**
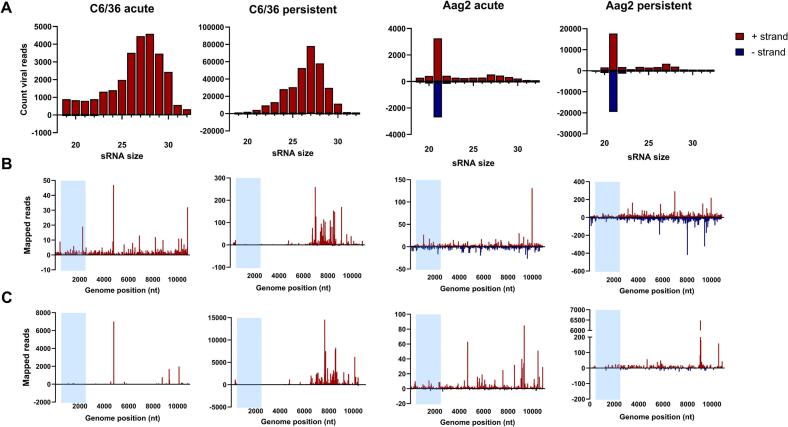
Small RNA response to BinJ-ZIKV during acute or persistent infection in C6/36 and Aag2 cells. **(A)** Size distribution of 19–30 nt small RNAs that map to the genome of BinJ-ZIKV. **(B)** Genome distribution of 21 nt small RNAs that map to the genome of BinJ-ZIKV. **(C)** Genome distribution of 25–30 nt small RNAs that map to the genome of BinJ-ZIKV. Blue square indicates the location of the ZIKV prME genes. (For interpretation of the references to colour in this figure legend, the reader is referred to the web version of this article.)

**Supplementary Fig. 2:**
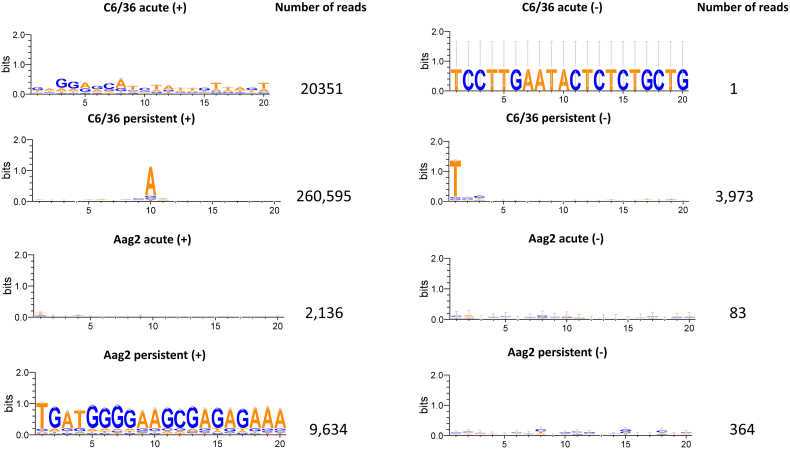
**Nucleotide biases of the 25-30 nt long RNAs of the acute and persistent BinJ-ZIKV infection in C6/36 and Aag2 cells.** Sequences are in DNA format, meaning U is written as T. Sense reads are placed on the left, antisense reads on the right. The overrepresented sequence found in the Aag2 persistent sense reads can be contributed to a single high hotspot read found at nucleotide position 9065 to which 67% (6481) of the reads mapped. The overrepresented sequence found in the C6/36 acute antisense reads can be contributed to the fact that only one read was found indicating that the production of antisense reads was very limited.

As was expected, no 21 nt peak was observed in the infected Dcr-2-deficient C6/36 cells (Fig. 5A, left), with only a small portion of the 21 nt reads mapping to the sense (+) strand of the BinJ-ZIKV genome and almost no 21 nt reads mapping to the antisense (−) strand (Fig. 5B, left). However, in both the acute and persistent infection a clear shoulder of 25–30 nt small RNAs was observed (Fig. 5A, left) that mapped only to the sense strand (Fig. 5C, left). This shoulder is indicative of viral piRNAs and was also more prominent in the persistent infection compared to the acute infection, despite the lower titers in these cells. The 25–30 nt shoulder in both C6/36 infections was analyzed for 1 U or 10 A biases, which is a characteristic signature of the ping-pong cycle and is indicative of ping-pong amplified viral piRNA production [29,30]. No such biases were found in the acute infection of C6/36. Remarkably, a clear A10 (+) and 1 U (−) bias was observed in the sequences of the 25–30 nt long RNAs that mapped to the sense strand in the persistently infected C6/36 cells (Fig. S2).

In contrast to the C6/36 cells, both persistently and acutely infected Aag2 cells mounted a potent siRNA response to BinJ-ZIKV as a 21 nt peak was observed that mapped to both the sense and antisense strand. Interestingly, more 21-nt reads mapped to the genome of BinJ-ZIKV in the persistent infection than in the acute infection (Fig. 5A, right), despite lower titers. Mapping of the 21 nt reads to the genome of BinJ-ZIKV demonstrated that the siRNAs targeted the entire genome in both the acute and persistent infection even though some hot and cold spots could be distinguished (Fig. 5B, right). Mapping of the 25–30 nt small RNAs to the genome of BinJ-ZIKV showed that these small RNAs almost exclusively mapped to the sense strand for both the acute and persistent Aag2 infections. However, compared to infections in C6/36 cells, only a low number of 25–30 nt reads from Aag2 cells mapped to the BinJ-ZIKV genome (Fig. 5C, right) and no 1 U or 10 A bias was observed for the 25–30 nt small RNAs that originated from the acute Aag2 cells (Fig. S2). In persistently infected Aag2 cells we observed a 10 A bias without 1 U bias (Fig. S2). However, this dataset was skewed by a single highly predominant sequence mapping to position 9065 of the BinJ-ZIKV genome.

### 3.5 High CPE in RNAi-defective mosquito cell lines upon BinJV and BinJ-ZIKV infection correlates with SIE

**Supplementary Fig. 3:**
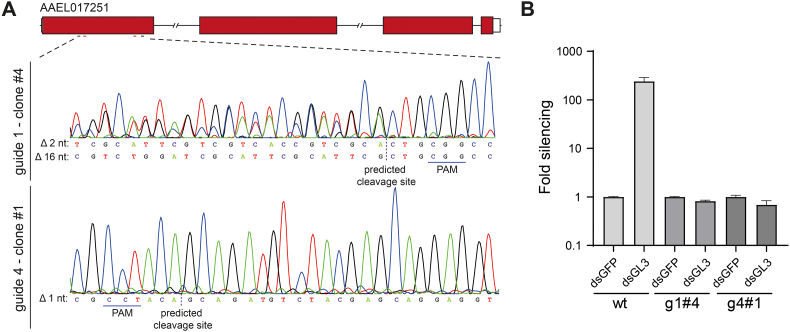
**Argonaute 2 knockout in Aag2 cells**. **(A)***Ago2* knockout cells were generated CRISPR/Cas9-mediated editing of the *Ago2* gene (AAEL017251) in Aag2 C3PC12 cells, single-cell colonies were grown, and the edited sites were Sanger sequenced. Top panel, Ago2 gene structure. Bottom panel, Sanger sequencing identified two cell clones with out-of-frame mutations in the first exon of *Ago2*. The PAM sequence and predicted cleavage site are indicated. **(B)** RNAi efficiency in luciferase based reporter assays in two Ago2 knockout clones (g1#4 and g4#1) and parental wildtype (wt) cells. Cells were transfected with plasmids encoding firefly luciferase (Fluc) and *Renilla* luciferase (Rluc) along with dsRNA targeting Fluc (dsFluc) or GFP (dsGFP) as a control. The Fluc counts were normalized to Rluc counts and the fold silencing was calculated relative to the dsGFP control for each cell line. Bar graphs represent means and standard deviation of 3 replicates. Absent silencing in the *Ago2* knockout clones confirms that these cells are RNAi deficient. Clone g4#1 has been used for infection experiments.

To further investigate whether the observed high SIE and CPE in C6/36 cells upon BinJV or BinJ-ZIKV infection was due to the lack of a functional RNAi response, we generated Argonaute2 (Ago2) deficient Aag2 cells using CRISPR/Cas9 technology (Fig. S3). Ago2 is a crucial protein involved in the siRNA response of insects as it uses siRNAs as a guide to detect and cleave complementary RNAs [31]. Therefore, similar to C6/36 cells, Aag2 Ago2-deficient cells also display a dysfunctional RNAi response, incapable of cleaving the (viral) target RNAs (Fig. S3).

**Fig. 6:**
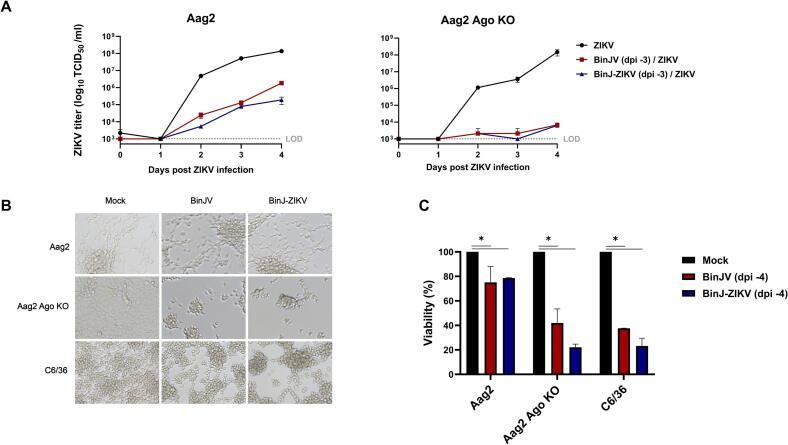
Superinfection with ZIKV of BinJ-ZIKV infected Aag2 Ago2-deficient (KO) cells. **(A)** ZIKV replication in Aag2 or Aag2 Ago2-deficient cells acutely (MOI 1) infected (3 days before ZIKV infection) with BinJV or BinJ-ZIKV. Supernatant was collected on the indicated days and viral titers were determined using EPDA. Shown are the mean virus titers and SEM from 3 biological replicates. The limit of detection (LOD) for ZIKV, WNV and CHIKV was 1 × 10^3^ TCID_50_/ml. **(B)** Brightfield pictures of Aag2, Aag2 Ago2-deficient and C6/36 cells mock-infected or acutely infected with BinJV or BinJ-ZIKV (MOI 1, 4 dpi). **(C)** MTT assay measuring the viability of Aag2, Aag2 Ago2-deficient or C6/36 cells mock-infected and acutely infected with BinJV or BinJ-ZIKV (MOI 1, 4 dpi). Shown are the mean viability and SEM from 3 biological replicates. Uninfected cells were set at 100 % viability and data is presented relative to this control. Asterisks indicate significant differences (*p* < 0.05, one-way ANOVA) between samples.

Wildtype (WT) and Ago2-deficient Aag2 cells were seeded and infected with BinJV and BinJ-ZIKV. Three dpi, uninfected Aag2 and Aag2 Ago2-deficient cells were density matched with the infected cells and all cells were infected with ZIKV. In cells that were not infected with BinJV or BinJ-ZIKV, ZIKV grew to similar titers in both cell types (1.4 × 10^8^ TCID50/ml and 1.5 × 10^8^ TCID50/ml at 4 days post ZIKV infection for Aag2 and Ago2-deficient Aag2, respectively) (Fig. 6A). ZIKV replication was significantly decreased at 4 days post ZIKV infection (*p* < 0.0001) in WT Aag2 cells infected with either BinJV or BinJ-ZIKV compared to ZIKV infections in cells not previously infected with BinJV or BinJ-ZIKV. Moreover, the BinJ-ZIKV infected Aag2 cells displayed lower ZIKV titers compared to BinJV-infected Aag2 cells with a 10-fold reduction in titer (Fig. 6A), similar to what was observed in Fig. 4.

**Supplementary Fig. 4:**
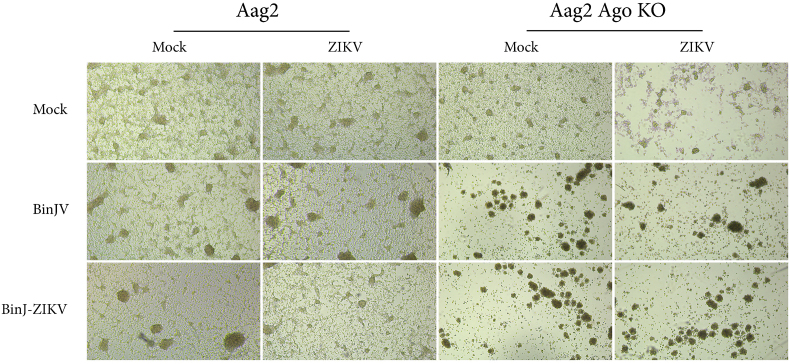
**Aag2 Ago KO cells displaying CPE upon BinJV,  Binj-ZIKV and ZIKV infection**. Brightfield pictures of Aag2 and Aag2 Ago2 KO cells at 7 days post BinJV or BinJ-ZIKV (MOI 1) infection and 4 days post ZIKV infection (MOI 1).

Interestingly, 4 days post BinJV/BinJ-ZIKV infection and 1 day post ZIKV superinfection, Aag2 Ago2-deficient cells infected with either BinJV or BinJ-ZIKV started to show CPE (Fig. 6B). At 7 days post BinJV/BinJ-ZIKV and 4 days post ZIKV superinfection Aag2 Ago2-deficient cells showed severe CPE, with most cells having detached from the bottom of the plate and clumped together. In contrast, the WT Aag2 cells displayed no observable CPE (Fig. S4).

ZIKV replication was severely impacted in the Aag2 Ago2-deficient cells that were acutely infected with either BinJV or BinJ-ZIKV. ZIKV titers were just above the LOD and similar for both the BinJV and BinJ-ZIKV infected Aag2 Ago2-deficient cells reaching 6.3 × 10^3^ TCID_50_/mL at 4 dpi (Fig. 6A). The severe CPE observed in infected Aag2 Ago2-deficient cells prohibited the generation of persistently infected cell lines and made it difficult to accurately assess whether the lack of Ago2 would abolish the observed difference in SIE between ZIKV on BinJV and BinJ-ZIKV infected cells.

To test whether a dysfunctional siRNA response affects cell viability during BinJV or BinJ-ZIKV infection, the viability of normal and Ago2-deficient Aag2 cells and C6/36 cells was measured in a dimethyl thiazolyl diphenyl tetrazolium salt (MTT) assay. All three cell types were either infected with BinJV or BinJ-ZIKV (MOI 1) and their viability compared to uninfected controls at 4 dpi. Although no CPE was observed in the WT Aag2 cells (Fig. 6B), a significant drop in viability of approximately 25 % was measured at 4 dpi for both the BinJV and BinJ-ZIKV infected cells (*p* < 0.05) (Fig. 6C). In accordance, the infected Aag2 Ago2-deficient cells displayed severe CPE at 4 dpi with cells clumping and detaching from the monolayer, resulting in a markedly reduced confluency compared to the mock-treated cells. Moreover, the viability of these infected Aag2 Ago2-deficient cells was significantly lower compared to WT Aag2 cells, displaying 60 % and 80 % reductions in viability after infections with BinJV and BinJ-ZIKV, respectively (*p* < 0.01) (Fig. 6B,C). In all our experiments, C6/36 cells also displayed strong CPE 4 dpi with BinJV and BinJ-ZIKV, with cell clumping and a reduced confluency of the monolayer compared to the mock-treated cells. Similar to the situation in Aag2 Ago2-deficient cells, the viability of C6/36 was reduced by 60 % and 80 % after infections with BinJV and BinJ-ZIKV, respectively (*p* < 0.0001) (Fig. 6B,C). Taken together, these results show that the viability of cells without a functional siRNA pathway is severely impacted by BinJV infection and suggest that reduced cell viability contributes strongly to the observed reduced replication of subsequent superinfection with arboviruses.

## 4 Discussion

In this study we have generated four persistently infected cell lines, namely Aag2 and C6/36 cells infected with either BinJV or BinJ-ZIKV to create a more representative model of SIE. These persistent infections were compared to acute infections to obtain a better understanding of *in vitro* SIE between dISFs and arboviruses.

Infectious virus was consistently detected in the cell-culture supernatant for all four combinations of cell line and virus during at least 65 passages. After 10 to 30 passages, depending on the original cell line used, the acute phase of the infections subsided and all cell lines reached a stable phase of infection maintaining low-level virus replication (Fig. 1). This observation is consistent with the generation of a C6/36 cell line persistently infected with Japanese encephalitis virus (JEV) where virus levels declined after the acute infection but remained stable for at least 35 passages [32]. Interestingly, in these persistently infected JEV cells defective viral RNAs with deletions in the E gene were detected already after one passage. Similarly, during the establishment of C6/36 persistently infected with DENV serotype 2 (DENV-2), defective viral genomes (DVGs) with deletions in the E and NS5 gene were also detected [33]. After 42 passages, infectious DENV-2 could no longer be detected by plaque assay in BHK cells, but viral sequences were detectable by RT-PCR. This is in contrast with our observations where during all passages the supernatant of all persistently infected cell lines caused strong CPE in naive C6/36 as observed during EPDAs. Although we have not specifically investigated the generation of DVGs in our persistent cell lines, the small RNA-seq analysis of infected Aag2 cells showed that 21 nt siRNAs mapped very evenly across the entire BinJ-ZIKV genome, indicating no deletions had occurred (Fig. 5). For C6/36 it is difficult to determine from the small RNA-seq data whether defective viral RNAs were generated as C6/36 only produced piRNAs for which it is common that reads do not map to the entire genome, but rather to specific hotspots in the genome of flaviviruses [18,34].

The ability of ISFs to interfere with the replication of medically important flaviviruses in mosquito cells has been reported before [[4], [5], [6], [7], [8],^35^]. The majority of the *in vitro* studies have been performed in the C6/36 cell line and with acute infections [7,8,35]. Similar to these studies we found that BinJV significantly reduced the replication of WNV and ZIKV during acute infection in C6/36 cells with a reduction in titers to below the LOD. In addition, when C6/36 cells were persistently infected with BinJV the reduction in viral titers for WNV and ZIKV was less prominent than for cells acutely infected with BinJV (Fig. 3). CHIKV replication was not inhibited at all by a persistent BinJV infection in C6/36 cells. This suggests that the observed CPE in C6/36 infected with BinJV (Figs. 1A, 6B,C and supplement S1) may cause the restricted replication of CHIKV and enhance SIE of ZIKV and WNV. This is supported by the observation that in Nhumirim virus (NHUV) infected C6/36 cells CPE is apparent from 3 dpi onwards [8]. The reported reduction in CHIKV replication was only observed when cells were infected at 3 dpi with NHUV and not when CHIKV and NHUV were co-inoculated on non-infected C3/36 cells. This suggests that during co-inoculation, CHIKV encounters viable cells, unlike in consecutive infection where cells already display CPE. Thus, the observed SIE in acute ISF infections that cause CPE in C6/36 cells may result from poor cell viability rather than direct ISF replication. In an effort to strengthen this hypothesis and confirm that the lack of an siRNA response in C6/36 is what causes these cells to develop CPE in response to ISF infections, we created a second siRNA deficient cell line, the Ago2-deficient Aag2 cells which lack functional Ago2 protein as opposed to Dcr2 in C6/36 (Fig. S3). In contrast to Dcr2 deficient C6/36 cells, we were unsuccessful in generating persistently infected Ago2-deficient Aag2 cells, which might suggest differential roles for Dcr2 and Ago2 in cellular responses to infection. Ago2-deficient Aag2 cells showed severe CPE at 4 dpi with BinJV or BinJ-ZIKV which strongly reduced ZIKV replication during superinfection. ZIKV titers were also reduced in the wild type Aag2 cells acutely infected with BinJV or BinJ-ZIKV, but this effect was less pronounced (Fig. 6A). The cell viability assay also showed that the Aag2 cells without a functional RNAi response developed strong CPE and eventual cell death upon infection of BinJV or BinJ-ZIKV, similar to infections in C6/36 cells (Fig. 6C). In contrast, the WT Aag2 cells with a functional RNAi response did not exhibit CPE upon infection and had a higher viability compared to the RNAi-deficient cell lines. Therefore, C6/36 cells or other cell lines with a defective RNAi response are a poor *in vitro* model for virus infections in mosquitoes, and specifically ISF infections and SIE experiments.

RNAi is a potent antiviral mechanism that generates viral siRNAs to degrade cognate RNA in virus infected cells (reviewed in [9]). Acute and persistent infection with BinJ-ZIKV in Aag2 cells resulted in detectable siRNAs that mapped to the ZIKV prME (Fig. 5). Subsequent infection with ZIKV resulted in reduced ZIKV replication compared to ZIKV infections in BinJV infected cells. This effect was not observed for superinfection with WNV, likely because the siRNAs generated from dsRNA intermediates of BinJ-ZIKV lack the sequence homology to target the WNV genome. Therefore, we conclude that the observed increase in SIE in RNAi competent Aag2 cells (Fig. 6) is the result of sequence homology between BinJ-ZIKV and ZIKV and governed by an antiviral siRNA response. This observation is further supported by a study in which ZIKV DVGs were used to transfect C6/36 and U4.4 (*Aedes albopictus* cells with a functional RNAi system) cells which were subsequently challenged with ZIKV, and where SIE was observed in the U4.4 cells but not in C6/36 [36]. Similar results were obtained for the SIE of CHIKV by CHIKV DVGs [37], thus indicating that sequence homology promotes SIE through RNAi. However, it should be noted that most Aag2 cells are persistently infected with insect-specific viruses including the flavivirus CFAV and phenuivirus PCLV. Although the Aag2 cells used in this study were previously cleared from PCLV and CFAV [23], hits mapping to parts of the PCLV genome were found during the sRNA-seq analysis (data not shown). The Ago2-deficient Aag2 cell line was derived from this cell line and as persistent virus infections can be lost when clonal cell lines are created [38], potential differences between both viromes may exist.

Taken together, our results show that SIE can be dissected into various components that contribute to its overall effect, which in different cell lines manifests through distinct mechanisms. As discussed above, sequence homology between two viruses can stimulate an RNAi response and in C6/36 cells and other cell lines with a dysfunctional RNAi system, the CPE caused by the ISF plays a significant role in the observed SIE. However, not all observed SIE can be attributed to CPE or RNAi as persistently infected C6/36 cells displaying no discernible CPE cause lower, but still significant levels of SIE. In general, the molecular mechanisms underlying superinfection exclusion are hypothesized to involve reduced receptor binding and impaired viral entry, and/or competition for cellular resources, leading to decreased RNA replication and translation of the secondary virus [39]. For NHUV it has been reported that SIE was likely not established *via* impaired viral entry and the authors hypothesized that SIE mostly occurs between closely related viruses which need to compete for similar host cell resources, as NHUV had a very limited impact on CHIKV replication as opposed to DENV and ZIKV [5]. In our study we found similar results in C6/36 cells, where CHIKV did not seem to be affected by a persistent BinJV infection. However, in Aag2 cells we did observe SIE of CHIKV by BinJV in both the acute and persistent infections. These findings suggest that SIE not only varies depending on the type of virus but also on the cell lines, highlighting the importance of using multiple cell lines in *in vitro* SIE research and knowing the activity of their specific antiviral pathways. While these *in vitro* results provide valuable insights, further *in vivo* research is crucial to fully understand the dynamics of SIE in natural environments. Importantly, initial *in vivo* studies have demonstrated that ISFs can affect the replication and transmission of arboviruses in mosquito vectors [4,6,40,41]. However, further investigations are needed to elucidate how mosquito colonies become persistently infected with ISFs and whether this affects the level of SIE compared to intrathoracic injections of ISFs.

In conclusion, our study demonstrates that SIE is a complex, multifaceted phenomenon influenced by various factors, including cell line characteristics, the functionality of the RNAi response, and sequence homology between viruses. The use of C6/36 cells for studying SIE seems therefore less suitable as their lack of a functional RNAi system does not accurately reflect biological processes that occur *in vivo.* Moreover, we also find that the CPE caused by BinJV in these cells can lead to exaggerated or misleading results, further complicating the interpretation of SIE dynamics. As many ISFs are known to cause CPE in C6/36 cells, using this cell line for studying SIE is inadvisable. Therefore, for the *in vitro* study of SIE, the use of RNAi-competent cells is recommended, as it more accurately reflects *in vivo* conditions and can uncover SIE driven by sequence homology. Finally, while *in vitro* studies are crucial for initial screening and mechanistic insides, *in vivo* research remains essential for uncovering the true impact of ISFs on MBFVs.

The following are the supplementary data related to this article.

Supplementary material R code used for the generation of size distributions of small RNAs, genome distributions of siRNAs and piRNAs and 3’trimming of mapped sense and reverse-complemented antisense reads of length 25-30 nt. Supplementary material

## Funding

Wessel Willemsen was funded by a personal grant from the Graduate School Production Ecology & Resource Conservation. Dr. Jelke Fros is supported by a VIDI grant from the Dutch Research Council (NWO; VI. Vidi. 213.027).

## CRediT authorship contribution statement

**Wessel Willemsen:** Writing – original draft, Visualization, Validation, Methodology, Investigation, Funding acquisition, Formal analysis, Data curation, Conceptualization. **Nick Helmes:** Methodology, Investigation. **Gijs J. Overheul:** Validation, Methodology, Investigation, Formal analysis. **Marleen Henkens:** Investigation. **Ruben Spruijt:** Methodology, Investigation. **Ronald P. van Rij:** Writing – review & editing, Supervision, Resources. **Monique M. van Oers:** Writing – review & editing, Supervision, Project administration. **Gorben P. Pijlman:** Writing – review & editing, Supervision, Funding acquisition, Conceptualization. **Jelke J. Fros:** Writing – review & editing, Supervision, Project administration, Funding acquisition, Data curation, Conceptualization.

## Declaration of generative AI and AI-assisted technologies in the writing process

During the preparation of this work the author(s) used ChatGPT in order to improve language and readability. After using this tool/service, the author(s) reviewed and edited the content as needed and take(s) full responsibility for the content of the publication.

## Declaration of competing interest

The authors declare that they have no known competing financial interests or personal relationships that could have appeared to influence the work reported in this paper.

## Data Availability

Small RNA sequencing libraries have been deposited in the NCBI Sequence Read Archive (SRA) under the associated BioProjectID: PRJNA1201772.
