# Supplementary material for: Differential effect of acute versus persistent insect-specific flavivirus infection on superinfection exclusion of West Nile, Zika and chikungunya viruses in RNAi-competent and -deficient mosquito cells
Source: One Health. 2024 Dec 24;20:100960. doi: 10.1016/j.onehlt.2024.100960 (PMC11750571; doi:10.1016/j.onehlt.2024.100960)
Supplement: Supplementary material — R code used for the generation of size distributions of small RNAs, genome distributions of siRNAs and piRNAs and 3’trimming of mapped sense and reverse-complemented antisense reads of length 25-30 nt. [file mmc5.pdf]

```r
workwd <- setwd(<YOUR_FILE_PATH>)
figures.drive <- setwd(<YOUR_FILE_PATH>)

# CRAN packages
install.packages("tidyverse")
install.packages("openxlsx")

# Bioconductor packages
if (!requireNamespace("BiocManager", quietly = TRUE)) {
    install.packages("BiocManager")
}
BiocManager::install("sangeranalyseR")
BiocManager::install("Biostrings")


library("tidyverse")
library("sangeranalyseR")
library(dplyr)
library(ggplot2)
library(openxlsx)
library(Biostrings)

bases <- c("C" = "#56B4E9","G"="#F0E442", "T" = "#009E73", "A" = "#D55E00", "N" =
"grey")
colour.FLAG <- c("antisense" = "#009E73", "sense" = "#0072B2")

############################################################################
###   Small RNA sequencing - processing reads mapped in Galaxy   ###########
############################################################################

#Reads are mapped to viral genome using Bowtie2 in Galaxy and saved as SAM files.
#Positive (sense) strand mapped reads get FLAG code 0, negative (antisense)
mapped reads get FLAG code 16. Unmapped reads have code 4.
#For each sample two SAM files were generated in Galaxy, one with the positive
(_sense) reads and the other with the negative (_antisense) reads.

APZ_BinJV_ZIKV_antisense <- read.delim("path/to/your/file.sam", header = TRUE,
row.names = NULL, sep = "")[,1:12]
APZ_BinJV_ZIKV_antisense <- mutate(APZ_BinJV_antisense, Cell_line = "Aag2",
Treatment = "APZ")
colnames(APZ_BinJV_ZIKV_antisense) <- c("QNAME", "FLAG",  "RNAME", "POS", "MAPQ",
"CIGAR", "MRNM", "MPOS","ISIZE", "SEQ", "QUAL",  "OPT", "Cell_line", "Treatment")

APZ_BinJV_ZIKV_sense <- read.delim("path/to/your/file.sam", header = TRUE,
row.names = NULL, sep = "")[,1:12]
```

```
APZ_BinJV_sense_ZIKV <- mutate(APZ_BinJV_sense, Cell_line = "Aag2", Treatment =
"APZ")
colnames(APZ_BinJV_sense_ZIKV) <- c("QNAME", "FLAG",  "RNAME", "POS", "MAPQ",
"CIGAR", "MRNM", "MPOS","ISIZE", "SEQ", "QUAL",  "OPT", "Cell_line", "Treatment")

AZ_BinJV_ZIKV_antisense <- read.delim("path/to/your/file.sam", header = TRUE,
row.names = NULL, sep = "")[,1:12]
AZ_BinJV_ZIKV_antisense <- mutate(AZ_BinJV_antisense, Cell_line = "Aag2",
Treatment = "AZ")
colnames(AZ_BinJV_ZIKV_antisense) <- c("QNAME", "FLAG",  "RNAME", "POS", "MAPQ",
"CIGAR", "MRNM", "MPOS","ISIZE", "SEQ", "QUAL",  "OPT", "Cell_line", "Treatment")

AZ_BinJV_sense <- read.delim("path/to/your/file.sam", header = TRUE, row.names =
NULL, sep = "")[,1:12]
AZ_BinJV_sense <- mutate(AZ_BinJV_sense, Cell_line = "Aag2", Treatment = "AZ")
colnames(AZ_BinJV_ZIKV_sense) <- c("QNAME", "FLAG",  "RNAME", "POS", "MAPQ",
"CIGAR", "MRNM", "MPOS","ISIZE", "SEQ", "QUAL",  "OPT", "Cell_line", "Treatment")

CPZ_BinJV_antisense <- read.delim("path/to/your/file.sam", header = TRUE,
row.names = NULL, sep = "")[,1:12]
CPZ_BinJV_antisense <- mutate(CPZ_BinJV_antisense, Cell_line = "C636", Treatment
= "CPZ")
colnames(CPZ_BinJV_ZIKV_antisense) <- c("QNAME", "FLAG",  "RNAME", "POS", "MAPQ",
"CIGAR", "MRNM", "MPOS","ISIZE", "SEQ", "QUAL",  "OPT", "Cell_line", "Treatment")

CPZ_BinJV_ZIKV_sense <- read.delim("path/to/your/file.sam", header = TRUE,
row.names = NULL, sep = "")[,1:12]
CPZ_BinJV_ZIKV_sense <- mutate(CPZ_BinJV_sense, Cell_line = "C636", Treatment =
"CPZ")
colnames(CPZ_BinJV_ZIKV_sense) <- c("QNAME", "FLAG",  "RNAME", "POS", "MAPQ",
"CIGAR", "MRNM", "MPOS","ISIZE", "SEQ", "QUAL",  "OPT", "Cell_line", "Treatment")

CZ_BinJV_ZIKV_antisense <- read.delim("path/to/your/file.sam", header = TRUE,
row.names = NULL, sep = "")[,1:12]
CZ_BinJV_ZIKV_antisense <- mutate(CZ_BinJV_antisense, Cell_line = "C636",
Treatment = "CZ")
colnames(CZ_BinJV_ZIKV_antisense) <- c("QNAME", "FLAG",  "RNAME", "POS", "MAPQ",
"CIGAR", "MRNM", "MPOS","ISIZE", "SEQ", "QUAL",  "OPT", "Cell_line", "Treatment")

CZ_BinJV_ZIKV_sense <- read.delim("path/to/your/file", header = TRUE, row.names =
NULL, sep = "")[,1:12]
CZ_BinJV_ZIKV_sense <- mutate(CZ_BinJV_sense, Cell_line = "C636", Treatment =
"CZ")
colnames(CZ_BinJV_ZIKV_sense) <- c("QNAME", "FLAG",  "RNAME", "POS", "MAPQ",
"CIGAR", "MRNM", "MPOS","ISIZE", "SEQ", "QUAL",  "OPT", "Cell_line", "Treatment")
```

```r
data.file <- rbind(APZ_BinJV_ZIKV_antisense, APZ_BinJV_ZIKV_sense,
AZ_BinJV_ZIKV_antisense, AZ_BinJV_ZIKV_sense, CPZ_BinJV_ZIKV_antisense,
CPZ_BinJV_ZIKV_sense, CZ_BinJV_ZIKV_antisense, CZ_BinJV_ZIKV_sense)
colnames(data.file) <- c("QNAME", "FLAG",  "RNAME", "POS", "MAPQ", "CIGAR",
"MRNM", "MPOS","ISIZE", "SEQ", "QUAL",  "OPT", "Cell_line", "Treatment")

data.file <- mutate(data.file, RC = chartr("ATGC", "TACG", SEQ))

viral.reads.tmp <- data.file

save(viral.reads.tmp, file = "path/to/your/file_reads_tmp.RData")
load(file = "path/to/your/file_tmp.RData")

############################################################################
###          Size distribution of sense and antisense reads             ###
############################################################################

# This code is used to generate an excel file with the size distribution of the
sense and antisense reads

load(file = "path/to/your/file_tmp.RData")

reads_APZ <- nrow(filter(viral.reads, Treatment == "APZ"))
reads_AZ <- nrow(filter(viral.reads, Treatment == "AZ"))
reads_CPZ <- nrow(filter(viral.reads, Treatment == "CPZ"))
reads_CZ <- nrow(filter(viral.reads, Treatment == "CZ"))

#Plot the division showing the sense/antisense strand in color
data.plot <-  filter(viral.reads) %>%
  select(Treatment, Length, FLAG) %>%
  group_by(Treatment, FLAG, Length) %>%
  summarise(Count = n()) %>%
  mutate(Fraction_total = ifelse(Treatment == "APZ", Count/reads_APZ,
ifelse(Treatment == "AZ", Count/reads_AZ, ifelse(Treatment == "CPZ",
Count/reads_CPZ, Count/reads_CZ)))) %>%
  mutate(Fraction_for_plot = ifelse(FLAG == 16, Fraction_total*-1,
Fraction_total)) %>%
  mutate(Direction = ifelse(FLAG == 16, "antisense", "sense"))
data.plot <- as.data.frame(data.plot)
head(data.plot)


# Specify the file path to save the Excel file
excel_file <- "path/to/your/file.xlsx"
```

```
# Write the data to an Excel file
write.xlsx(data.plot, file = excel_file, sheetName = "Treatment_Position_Counts",
rowNames = FALSE)

############################################################################
###                    21nt genome distribution                          ###
############################################################################

# This code is used to generate an excel file that contains the 21nt reads that
mapped to your fasta file and gives the sense and antisense reads counts for each
nucleotide position on the genome

#Calculate total reads and divide in sense and antisense

load(file = "path/to/your/file_tmp.RData")

viral.reads <-  data.file %>%
  mutate(Length = nchar(SEQ)) %>%
  mutate(Position = ifelse(FLAG == 16, POS + Length, POS)) %>%
  mutate(Sequence = ifelse(FLAG == 16, RC, as.character(SEQ))) %>%
  mutate(First_nt = substr(Sequence, 0, 1)) %>%
  mutate(Last_nt = substr(Sequence, nchar(Sequence), nchar(Sequence)))
head(viral.reads)

save(viral.reads, file = "path/to/your/file.RData")
load(file = "path/to/your/file.RData")

# Filter reads with Length == 21
reads_length_21 <- filter(viral.reads, Length == 21)

# Define a function to calculate position counts for a specific treatment
calculate_position_counts <- function(treatment) {
  # Filter reads for the specified treatment
  treatment_reads <- filter(reads_length_21, Treatment == treatment)

  # Separate reads into sense (FLAG == 0) and antisense (FLAG == 16)
  sense_reads <- filter(treatment_reads, FLAG == 0)
  antisense_reads <- filter(treatment_reads, FLAG == 16)

  # Count positions for sense reads
  sense_position_counts <- count(sense_reads, Position)

  # Count positions for antisense reads
  antisense_position_counts <- count(antisense_reads, Position)
```

```
  # Merge sense and antisense counts by Position
  position_counts <- full_join(sense_position_counts, antisense_position_counts,
by = "Position", suffix = c("_sense", "_antisense")) %>%
    mutate(count_sense = ifelse(is.na(n_sense), 0, n_sense),
           count_antisense = ifelse(is.na(n_antisense), 0, n_antisense)) %>%
    select(Position, count_sense, count_antisense)

  return(position_counts)
}

# List of treatments
treatments <- c("APZ", "AZ", "CPZ", "CZ")

# Create a list to store position counts for each treatment
position_counts_list <- lapply(treatments, calculate_position_counts)

# Combine position counts for all treatments into a single data frame
combined_position_counts <- bind_rows(Map(cbind, treatment = treatments,
position_counts_list))

# Specify the file path to save the Excel file
excel_file <- "path/to/your/file.xlsx"

# Write the data to an Excel file
write.xlsx(combined_position_counts, file = excel_file, sheetName =
"Treatment_Position_Counts", rowNames = FALSE)

# Print a message indicating the completion of the export
cat("Position counts for each treatment have been exported to:", excel_file,
"\n")

#############################################################################
###                    25-30 nt genome distribution                       ###
#############################################################################

# This code is used to generate an excel file that contains the 25-30 nt (PiWi)
reads that mapped to your fasta file and gives the sense and antisense reads
counts for each nucleotide position on the genome

load(file = "path/to/your/file_tmp.RData")

# Filter reads with Length == 25-30
reads_length_25_30 <- filter(viral.reads, Length >= 25 & Length <= 30)
```

```r
# Define a function to calculate position counts for a specific treatment
calculate_position_counts <- function(treatment) {
  # Filter reads for the specified treatment
  treatment_reads <- filter(reads_length_25_30, Treatment == treatment)

  # Separate reads into sense (FLAG == 0) and antisense (FLAG == 16)
  sense_reads <- filter(treatment_reads, FLAG == 0)
  antisense_reads <- filter(treatment_reads, FLAG == 16)

  # Count positions for sense reads
  sense_position_counts <- count(sense_reads, Position)

  # Count positions for antisense reads
  antisense_position_counts <- count(antisense_reads, Position)

  # Merge sense and antisense counts by Position
  position_counts <- full_join(sense_position_counts, antisense_position_counts,
by = "Position", suffix = c("_sense", "_antisense")) %>%
    mutate(count_sense = ifelse(is.na(n_sense), 0, n_sense),
           count_antisense = ifelse(is.na(n_antisense), 0, n_antisense)) %>%
    select(Position, count_sense, count_antisense)

  return(position_counts)
}

# List of treatments
treatments <- c("APZ", "AZ", "CPZ", "CZ")

# Create a list to store position counts for each treatment
position_counts_list <- lapply(treatments, calculate_position_counts)

# Combine position counts for all treatments into a single data frame
combined_position_counts <- bind_rows(Map(cbind, treatment = treatments,
position_counts_list))

# Specify the file path to save the Excel file
excel_file <- "path/to/your/file.xlsx"

# Write the data to an Excel file
write.xlsx(combined_position_counts, file = excel_file, sheetName =
"Treatment_Position_Counts", rowNames = FALSE)

# Print a message indicating the completion of the export
cat("Position counts for each treatment have been exported to:", excel_file,
"\n")
```

```r
##########################################################################
###                            U10 sense trimmer                       ###
##########################################################################

# This code is used to generate a fasta file of all 25-30 nt sense reads that are
3'trimmed to 20 nt. This file can be used in Weblogo3 for image creation

load(file = "path/to/your/file_tmp.RData")

# Define the treatments
treatments <- c("APZ", "AZ", "CPZ", "CZ")

# Define the directory path
directory_path <- "path/to/your/folder"

# Loop over each treatment
for (treatment in treatments) {
  # Filter sequences based on treatment, length criteria, and sense sequences
  filtered_data <- viral.reads %>%
    filter(Treatment == treatment &
             nchar(SEQ) >= 25 & nchar(SEQ) <= 30 &
             FLAG == 0)  # Consider only sense sequences

  # Get the number of sequences used
  num_sequences <- nrow(filtered_data)

  # Pull sequences directly as character vector
  filtered_sequences <- pull(filtered_data, SEQ)

  # Convert filtered sequences to DNAStringSet
  filtered_sequences_DNA <- DNAStringSet(filtered_sequences)

  # Trim sequences to 20 nucleotides from the 3' end
  trimmed_sequences <- substring(filtered_sequences_DNA, 1, 20)

  # Convert the trimmed sequences back to character vector
  trimmed_sequences_char <- as.character(trimmed_sequences)

  # Create a DNAStringSet object from the trimmed sequences
  msa_sequences <- DNAStringSet(unlist(trimmed_sequences_char))

  # Define the file name
  file_name <- paste("alignment_", treatment, "_sense.fasta", sep = "")
```

```r
  # Write the DNAStringSet object to a FASTA file in the specified directory
  writeXStringSet(msa_sequences, file = file.path(directory_path, file_name))

  # Display the number of sequences used for each file
  cat("For treatment", treatment, ":", num_sequences, "sequences were used.\n")
}


##############################################################################
###                         U10 antisense trimmer                          ###
##############################################################################

# This code is used to generate a fasta file of all 25-30 nt antisense reads that
are 3'trimmed to 20 nt. This file can be used in Weblogo3 for image creation

load(file = "path/to/your/file_tmp.RData")

# Define the treatments
treatments <- c("APZ", "AZ", "CPZ", "CZ")

# Define the directory path
directory_path <- "path/to/your/folder"

# Loop over each treatment
for (treatment in treatments) {
  # Filter sequences based on treatment, length criteria, and antisense sequences
  filtered_data <- viral.reads %>%
    filter(Treatment == treatment &
             nchar(SEQ) >= 25 & nchar(SEQ) <= 30 &
             FLAG == 16)  # Consider only antisense sequences

  # Get the number of sequences used
  num_sequences <- nrow(filtered_data)

  # Pull sequences directly as character vector
  filtered_sequences <- pull(filtered_data, SEQ)

  # Convert filtered sequences to DNAStringSet
  filtered_sequences_DNA <- DNAStringSet(filtered_sequences)

  # Make the reads reverse complement
  filtered_sequences_DNA <- reverseComplement(filtered_sequences_DNA)

  # Trim sequences to 20 nucleotides from the 3' end
  trimmed_sequences <- substring(filtered_sequences_DNA, 1, 20)
```

```r
  # Convert the trimmed sequences back to character vector
  trimmed_sequences_char <- as.character(trimmed_sequences)

  # Create a DNAStringSet object from the trimmed sequences
  msa_sequences <- DNAStringSet(unlist(trimmed_sequences_char))

  # Define the file name
  file_name <- paste("alignment_", treatment, "_antisense.fasta", sep = "")

  # Write the DNAStringSet object to a FASTA file in the specified directory
  writeXStringSet(msa_sequences, file = file.path(directory_path, file_name))

  # Display the number of sequences used for each file
  cat("For treatment", treatment, ":", num_sequences, "sequences were used.\n")
}
```
